# Prospective clinical study to evaluate the success and survival of two-piece zirconia implants: a single-center study. 12-month results

**DOI:** 10.1186/s40729-025-00621-x

**Published:** 2025-04-20

**Authors:** Marie-Elise Jennes, Insa Herklotz, Margarita Bessonova, Jeremias Hey, Florian Beuer

**Affiliations:** 1 Department of Prosthodontics, Charité-Universitätsmedizin Berlin, Corporate Member of Freie Universität Berlin, Humboldt-Universität zu Berlin, Geriatric Dentistry and Craniomandibular Disorders, Assmanshauser Straße 4-6, 14197 Berlin, Germany; 2 Private Dental Practise, Amalienpark 1, 13187 Berlin, Germany; 3 Private Dental Practise, Ährenweg 1, 32469 Petershagen, Germany; 4 Department of Prosthetic Dentistry, University School of Dental Medicine, Martin Luther University Halle-Wittenberg, Magdeburger Str. 16, 06112 Halle, Germany

**Keywords:** Ceramic implants, Zirconia, Two-piece implants, Implant fracture, Screw-retained restoration, Dental implants, Implant survival, PEKK abutment

## Abstract

**Purpose:**

To evaluate the survival and success rates of a novel two-piece zirconia implant system restored with screw-retained glass–ceramic crowns over 12 months, including assessment of bone levels, soft tissue parameters, and patient-reported outcome measures.

**Methods:**

Twenty-four patients received single two-piece zirconia implants (CERALOG^®^ Hexalobe) in healed sites. After a 6-month healing period, implants received provisional screw-retained crowns on PEKK temporary abutments, followed by definitive lithium disilicate crowns (IPS e.max Press) on PEKK abutments. Clinical and radiographic examinations were performed at implant placement, re-entry, definitive loading, and 12-month follow-up, along with patient-reported outcome measures.

**Results:**

The implant survival rate at 12 months was 60.9%. Nine implants were lost: two due to lack of osseointegration at re-entry, four due to mobility after loading, and three due to fractures in the coronal third after loading. Surviving implants showed stable soft tissue parameters with mean probing pocket depths of 2.7 ± 0.7 mm at 12 months. The mean distance from implant shoulder to first bone contact decreased from 1.9 ± 0.6 mm at loading to 1.4 ± 0.6 mm at 12 months. Patients with surviving implants reported high satisfaction scores (4.8 ± 0.4) for function and aesthetics.

**Conclusions:**

The two-piece zirconia implant system with screw-retained restorations showed unsatisfactory survival rates. The combination of ceramic implants with screw-retained prosthetic restorations may have contributed to the higher failure rates observed. As a pilot study with a relatively small sample size, these findings should be confirmed by larger multicenter studies to validate these preliminary results.

## Background

Dental implants are a common and scientifically well-proven treatment option to replace missing or compromised teeth. Since the beginning of oral implantology, titanium implants have been predominantly used. Due to their high biocompatibility and good mechanical properties, titanium implants have demonstrated good osseointegration properties [1] and high long-term success and survival rates across different indications [2–4].

Improved implant placement and loading protocols were developed to reduce the  invasiveness of treatment for the patient. The concept ‘one time one abutment’ showed improved clinical results [5]. With the Munich implant concept [6, 7], a digitized protocol was presented with financial benefits and a biologically advantageous, one-abutment/one-time approach with customized screw-retained, full-contour crowns or cemented crowns on custom abutments.

However, titanium implants may not be the optimal choice for all patients. In cases with thin mucosal biotype, high smile line or in the esthetic zone, the dark color of the titanium implants might discolor the mucosal tissue and compromise the esthetic outcome [8]. Furthermore, in case of soft tissue recession the dark implant shoulder might become visible. To address these esthetic limitations, alternative materials have been explored.

Ceramic implants have shown comparable biocompatibility properties as titanium [9, 10]. In particular, the yttrium-stabilized tetragonal polycrystalline zirconia (Y-TZP) exhibits improved mechanical properties, making it a potential suitable material for the fabrication of dental implants. An in-vitro study even showed zirconia surfaces with reduced plaque biofilm formation compared to titanium surfaces [11]. The first systems on the market were one-piece ceramic implants. From both surgical and prosthodontic perspectives, however, two-piece implants are often preferred. To date, only limited clinical data are available regarding success and survival of two-piece ceramic implants. Furthermore, the existing literature on two-piece zirconia implants predominantly focuses on systems using cemented abutments and prosthetic crowns cemented on these abutments [12–16]. Notably, there is a significant gap in research regarding the performance of two-piece zirconia implants restored with screw-retained prosthetic crowns. This distinction is crucial, as the method of prosthetic attachment can significantly influence implant biomechanics, stress distribution, and ultimately, survival rates.

This prospective, longitudinal, clinical single-center study was designed to prove the concept of the novel two-piece zirconia implants restored with screw-retained prosthetic glass–ceramic crowns. This approach eliminates the need for cement, potentially reducing the risk of peri-implant diseases associated with excess cement [17]. The primary objective was to assess the survival and success of the implants up to 12 months post-loading and at yearly follow-ups over 5 years. The secondary objectives included the change in bone level, changes in soft tissue and patient-reported outcome measures (PROMs)—all at 12, 24, 36,48, and 60 months post-loading.

## Methods

### Study design and study population

This was a prospective, longitudinal, open clinical single-center study performed at the department of prosthodontics, geriatric dentistry and craniomandibular disorders of the Charité University Berlin (Germany). The study adhered to the principles outlined in the Declaration of Helsinki for experiments involving human subjects [18] and followed the STROBE guidelines for observational studies. The study was registered with DKRS (Deutsches Register Klinischer Studien).

In this present study, generally healthy adults with single tooth gaps and natural adjacent mesial and distal teeth in maxilla and mandible meeting the inclusion criteria (Table 1), who are scheduled to receive an implant supported single tooth restoration were included. The opposing dentition had to be natural teeth or restored with a fixed restoration. Minor bone augmentation was allowed (e.g. autogenous bone harvested during drilling). Written informed consent was obtained from all participants. Explicit exclusion criteria can be seen in Table 1.

**Table 1:** Inclusion and exclusion criteria

Inclusion criteria:
1. Males and females with at least 18 years of age
2. Single tooth gaps at least 7.5 mm wide in maxilla or mandible (#1-#7 FDI) allowing the placement of a single implant. Adjacent mesial and distal tooth must be natural tooth
3. Adequate bone quality and quantity at the implant site to permit the insertion of a zirconia implant
4. Opposing dentition must be natural teeth or fixed restoration
5. If patients undergo a large bone augmentation (with autogenous bone block) a delay of 3.5 months must elapse before implantation
6. Patient has been informed of the follow-up visits and is willing to return to the clinical center for these follow-up visits
**Pre-surgical exclusion criteria:**
7. Free-end situations
8. Systemic disease that would interfere with dental implant therapy (e.g. uncontrolled diabetes)
9. Any contraindications for oral surgical procedures
10. Patients who smoke > 10 cigarettes per day or cigar equivalents, or who chew tobacco
11. Cardiovascular disease
12. Coagulation disorders (including taking anticoagulants)
13. Metabolic bone disease
14. Chemotherapy or radiation treatment
15. Chronic inflammation
16. Metabolic or systemic disorders associated with lesions and/or bone healing
17. The use of pharmaceutical products that block or modify bone healing
18. Uncontrolled para-functional diseases (bruxism, clenching or grinding of teeth)
19. Insufficient inter-arc gap
20. Intraoral infection
21. Insufficient coverage of the soft tissue
22. Disorders that impede the ability of patients to maintain adequate oral hygiene
23. Conditions or circumstances, in the opinion of the investigator, which would prevent completion of study participation or interfere with analysis of study results, such as history of non-compliance or unreliability
**Secondary exclusion criteria at implant surgery:**
24. Lack of primary stability of the implant
25. GBR during surgery. Only minor bone augmentation will be allowed (e.g. autogenous bone harvested during drilling)

The recruitment period was 12 months. The pre-treatment examination included the following procedures: standardized radiographs of the existing situation to determine bone quality, bone quantity and to plan the treatment modalities, cone-beam computer tomography (CBCT), standardized intraoral photographs of the situation prior to surgery, demographic and habit descriptions of the intended implant site, and oral hygiene assessments. All patients gave their informed consent to participate in this pilot study.

### Implants

A novel two-piece zirconia implant (CERALOG^®^ Hexalobe implant, ALTATEC GmbH Wimsheim Germany) supporting single crowns in the maxilla and mandible was used. The implants, prosthetic parts, and other devices used for the study were CE-marked and used within the indications recommended by the manufacturer. The implants were available in diameter 4.0 mm and in lengths 8-, 10-, and 12-mm. All implants were placed in healed sites.

#### Implant placement

Prior to surgery, radiographs were meticulously evaluated to confirm adequate bone height for implant placement. Computer-guided implant surgery was employed to achieve precise, prosthetically driven implant positioning. Due to the unavailability of a guide system for the Zirconia implant, a template-guided pilot drilling using the Camlog Guide System was planned. CBCT scans were obtained with standardized settings using the 3D Accuitomo 170 (J. Morita Corporation, Kyoto, Japan), for better superimposition cotton rolls were placed in the vestibulum. Intraoral scans of the pre-operative situation were obtained with a powder-free computer aided impressioning device (Trios3, 3shape, Copenhagen, Denmark). The CBCT 3D data set (DICOM) and the STL (Standard Tessellation Language) data from optical scans were imported into a planning software (SMOP, Swissmeda, Zurich, Switzerland) and superimposed. The implants were planned in the prosthetically ideal position, with subsequent verification of their correct placement within the available bone. The distance from the adjacent natural teeth had to be at least 1.5 mm. The surgical template was digitally designed and printed by a centralized CAD/CAM system (Dedicam, Camlog BioHorizon).

In general, the implant treatment was performed according to the Munich Implant Concept (MIC) whenever possible and according to the standard procedures used in the hospital. No additional invasive examinations were done. Radiographs were taken in accordance with the standard treatment protocol. Surgery was performed under local anesthesia. Prophylactic antibiotics were given 1 h before surgery. The implant site was prepared according to the manufacturer’s instructions. A flap was raised, and a template-guided pilot drilling was executed. For bone quality of D1 and D2, a cortical drill was used for the cortical bone portions prior to the equicrestal insertion of the implants. During implantation only minor bone augmentation was allowed (i.e. bone harvested during drilling). If primary stability was achieved, the impression was taken before suturing. Submerged healing was applied. The patients were instructed with the after surgical care of the treated area. Standardized radiographs and photographs were taken immediately post-surgery. Sutures were removed 7 to 14 days post-surgery.

#### Second stage surgery (re-entry)

After a healing period of 6 months, the implant site was re-opened and exposed. A temporary Polymethyl-methacrylate screw retained crown mounted on a polyetherketoneketone (PEKK) temporary abutment was placed. Standardized radiographs and photographs were taken before and after abutment/crown placement to document the hard and soft tissue situation.

#### Prosthesis placement

The final restoration was a screw-retained crown (IPS e.max Press, Ivoclar Vivadent AG, Schaan, Lichtenstein) mounted on a PEKK abutment, secured with a titanium abutment screw. This definitive prosthesis was delivered within 4 months after temporary restoration placement, both fabricated by the same dental technician to ensure continuity in design and fit. The day of definitive prosthesis delivery was defined as the baseline for subsequent measurements. Standardized radiographs and photographs were taken after abutment/crown placement to document the hard and soft tissue situation. Soft tissue parameters were evaluated and measured. PROMs were obtained by a questionnaire.

#### Follow-up

The primary objective was to determine the survival of the Hexalobe implants up to 12 months post-loading and at yearly follow-ups over 5 years. Secondary objectives included the change in bone level, changes in soft tissue, and documentation of PROMs—all at 12-, 24-, 36-, 48-, and 60-months post-loading. Additionally, the nature and frequency of adverse events was recorded over the whole study period. An overview of the assessment procedures can be seen in Table 2. An overview of the primary and secondary endpoints can be seen in Table 3. An implant was classified as 'surviving' if it remained in situ throughout the entire observation period, in accordance with the criteria established by Albrektsson et al. [19],  Complications were documented and classified into pain, mobility, radiolucency, infection, and others. Mean values and standard deviations were calculated for each parameter.

**Table 2:** .

		Visit 1	Visit 2	Visit 3	Visit 4	Visit 5	Visit 6	Visit 7	Visit 8	Visit 9	Visit 10
		Recruitment/Screening/IC	Implantation/Surgery/Impression	Suture removal	2nd stage surgery/re-entry	Definitive loading/reconstruction	12-month follow-up	24-month follow-up	36-month follow-up	48-month follow-up	60-month follow-up
Demographics	Informed consent (IC)	x									
	Demographic data	x									
General health assessment	Inclusion, exclusion (eligibility)		x								
	Health questionnaire	x									
	Change in patient's baseline		x	x	x	x	x	x	x	x	x
	Details opposing and adjacent dentition				x	x	x	x		x	
Dental history	Oral hygiene assessment	x	x	x	x	x	x	x	x	x	x
	Photographs	x			x	x	x	x	x	x	x
	Radiographs	x			x	x	x	x	x	x	x
	Implantation		x								
	Wound healing			x							
	Implant mobility/complications			x	x	x	x	x	x	x	x
	Oral hygiene instruction	x	x	x	x	x	x	x	x	x	x
	Clinical oral health	x	x	x	x	x	x	x	x	x	x
Treatment	Soft tissue clinical PPI/BOP/MGI/PPD					x	x	x	x	x	x
	PROMs					x	x	x	x	x	x
	Adverse events				x	x	x	x	x	x	x
	Concomitant medication	x	x	x	x	x	x	x	x	x	
Safety data	Study termination

**Table 3:** Primary and secondary endpoint parameters

Primary endpoint parameter
Implant survival and success [20] at 12 months post-loading Evaluation of any complications and adverse events
Secondary endpoint parameters
Assessment of bone level, at surgery, re-entry, prosthesis placement and at yearly follow-ups up to 5 years post-loading: Distance implant Shoulder to first bone contact at implant (DIB)
Evaluation of clinical parameters, at re-entry, prosthesis placement and at each follow-up up to 5 years post-loading: • Soft tissue clinical parameters, Plaque assessment (PPI), Bleeding on probing (BOP) [21], Modified gingival index (MGI) [22], Probing pocket depths (PPD) • Patient-reported outcome measures (PROMs) • Prosthetic outcome (AEs incl. chipping)

### Statistical evaluation

Descriptive statistics was performed with IBM SPSS Software (Version 29.0, Armonk, NY, USA). The data were analyzed for implant success and survival rates after 12 months.

## Results 12-month follow-up

Between April 2017 and March 2019, 24 single implants were placed in 24 generally healthy adult females (n = 11) and males (n = 13) with a mean age of 48.4 years (ranging between 27 and 77 years). One patient was a smoker (4.2%). The placed implants replaced 13 premolars and 11 molars. Thirteen implants were placed in the maxilla and 11 in the mandible. All implants had a diameter of 4.0 mm at the implant body and 4.5 mm at the implant shoulder. The intra-osseous lengths of the placed implants were 8 mm (n = 8) and 10 mm (n = 16). All implants showed primary stability after insertion. None of the placed implants showed peri-operative nor immediate post-operative complications up to the post OP examination (suture removal).

**Fig. 1:**
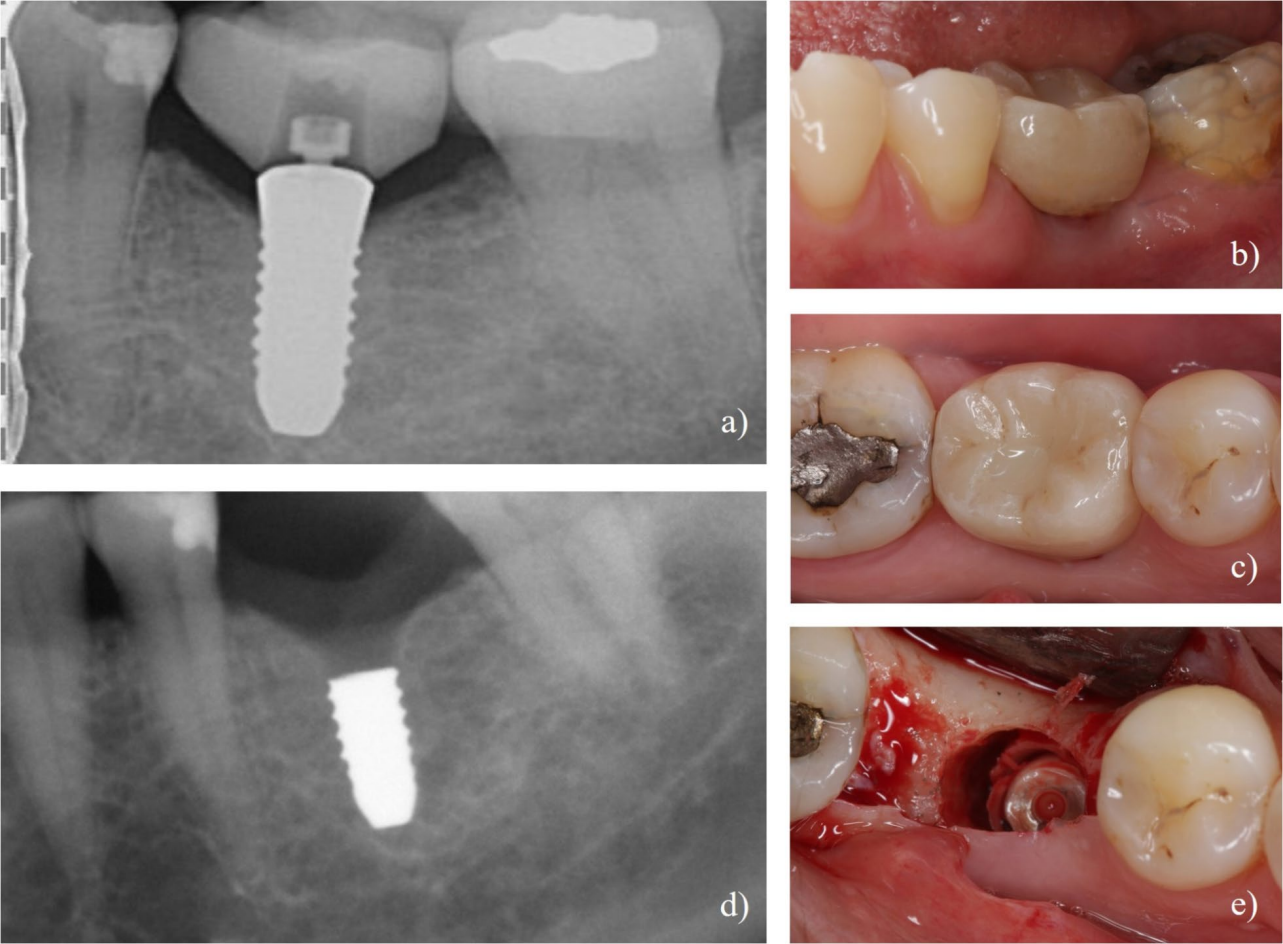
Case of implant fracture. **a** Initial radiograph showing dental implant with definitive screw retained crown and translucent PEKK abutment. **b**, **c** Clinical view of implant-supported crown in situ at time of definitive prosthesis placement. **d** Follow-up radiograph revealing fracture of coronal third of implant. **e** Clinical appearance after implant fracture, showing the fractured implant in situ

One patient (#24) did not present for any follow-up visits after the implantation, resulting in a study drop-out. Two implants were lost due to missing osseointegration and mobility before provisional loading at the time of reentry. Three implants failed after the provisional crown placement due to mobility (patient #8, #9, #16) and one implant (#3) due to fracture after provisional crown placement (Fig. 1).

**Fig. 2:**
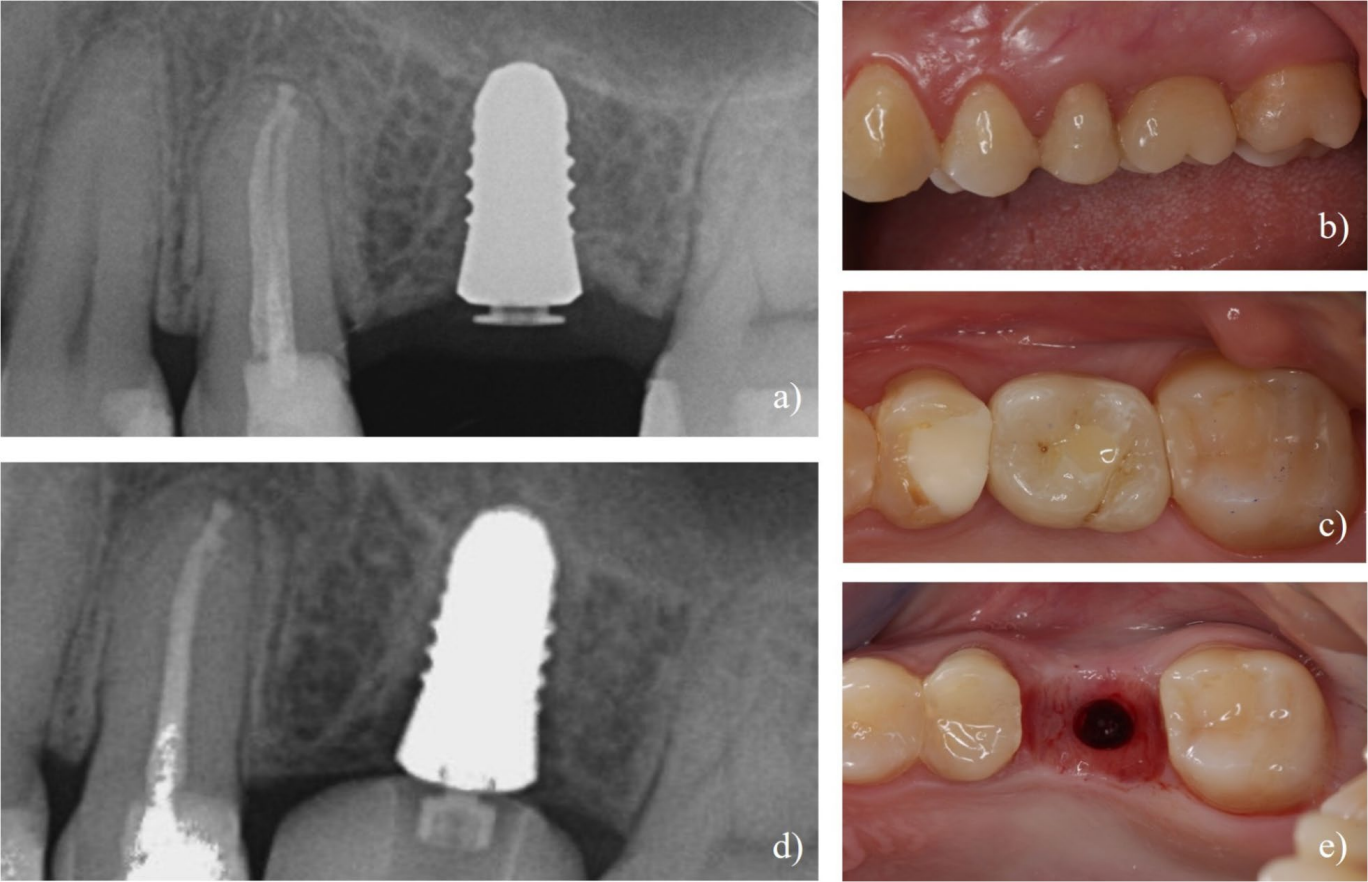
Case of implant loss due to implant mobility. **a** Initial radiograph showing dental implant with definitive screw retained crown and translucent PEKK abutment. **b**, **c** Clinical view of the implant-supported crown in situ at time of definitive prosthesis placement. **d** Follow-up radiograph revealing peri-implant radiolucency, indicative of implant failure due to loss of osseointegration. **e** Clinical appearance after implant removal, showing extraction socket

After definitive loading one implant was removed due to mobility (4 months after definitive loading in patient #6) Notably, all implants exhibiting mobility were characterized by a circumferential radiolucent area visible in the radiographic examinations (Fig. 2). Furthermore, two implants were lost due to fractures; these occurred in patient #17 two months after definitive loading, and patient #20 three months after definitive loading. All implants fractured at the coronal third (Fig. 2) and required additional surgery for removal of the apical implant part. Detailed information about the lost implants can be seen Table 4.

**Table 4:** Detailed information about lost implants

Patient no	Region	Gender	Reason of loss	Time of loss
#3	36	F	Fracture	After provisional crown placement
#6	26	F	Mobility	After definitive crown placement
#8	26	F	Mobility	After provisional crown placement
#9	24	M	Mobility	After provisional crown placement
#13	46	M	Mobility	At re-entry
#16	34	M	Mobility	After provisional crown placement
#17	25	M	Fracture	After definitive crown placement
#19	14	M	Mobility	At re-entry
#20	46	F	Fracture	After definitive crown placement

Overall, four of the lost implants were in the lower jaw, while five were in the upper jaw. All lost implants were replaced with titanium implants (Camlog Screw Line Promote Plus, Camlog BioHorizons) following a healing period of 3 month and prosthetic placement of screw-retained crowns. One patient reported pain as a biological complication for one month following the loading with a provisional crown. Notably, no pain was reported following the definitive loading. At time of definitive loading the survived implants showed a mean PPD of 2.4 ± 0.3 mm. Eleven sites (11.5%) exhibited PPD > 4 mm and only one site (1%) showed PPD > 5 mm. There were no occurrences of suppuration. The surviving implants showed an overall BOP of 17.7% at the implant sites. The clinical assessment of PPD and BOP of surviving implants at six sites is summarized in Table 5. The survival rate for the implants up to definitive loading was 73.9% (Table 6). The radiographic evaluation of marginal bone loss after definitive loading showed mean DIB values of 1.9 ± 0.6 mm.

**Table 5:** PPD (in mm), BOP of surviving implants at six sites and MGI at times of definitive loading, 12-month follow-up

	Definitive loading (n = 96 sites on 16 implants)	12-month follow-up (n = 66 sites on 11 implants)
Mean PPD in mm	2.4 ± 03 mm	2.7 ± 0.7 mm
PPD > 4 mm	11 (11.5%)	4 (6.1%)
PPD > 5 mm	1 (1%)	6 (9.1%)
BOP in %	17.7%	26%
	BOP > 0 = 5	BOP > 0 = 4
BOP	BOP > 1 = 6	BOP > 1 = 2
	BOP > 2 = 4	BOP > 2 = 1
	BOP > 3 = 1	BOP > 3 = 3
	BOP > 4 = 0	BOP > 4 = 1
	BOP > 5 = 0	BOP > 5 = 0
	BOP > 6 = 0	BOP > 6 = 0
Mean MGI	0.34 ± 0.46	0.38 ± 0.36
MGI	MGI > 0 = 75	MGI > 0 = 45
	MGI > 1 = 13	MGI > 1 = 17
	MGI > 2 = 7	MGI > 2 = 4
	MGI > 3 = 1	MGI > 3 = 0
	MGI > 4 = 0	MGI > 4 = 0

**Table 6:** Survival rates of two-piece zirconia implants at different time intervals

Time periode	Number of implants	Implants lost	% Survival
Placement to definitive loading	17	6	73.9%
Definitive loading to 12-month follow-up	14	3	60.9%

**Fig. 3:**
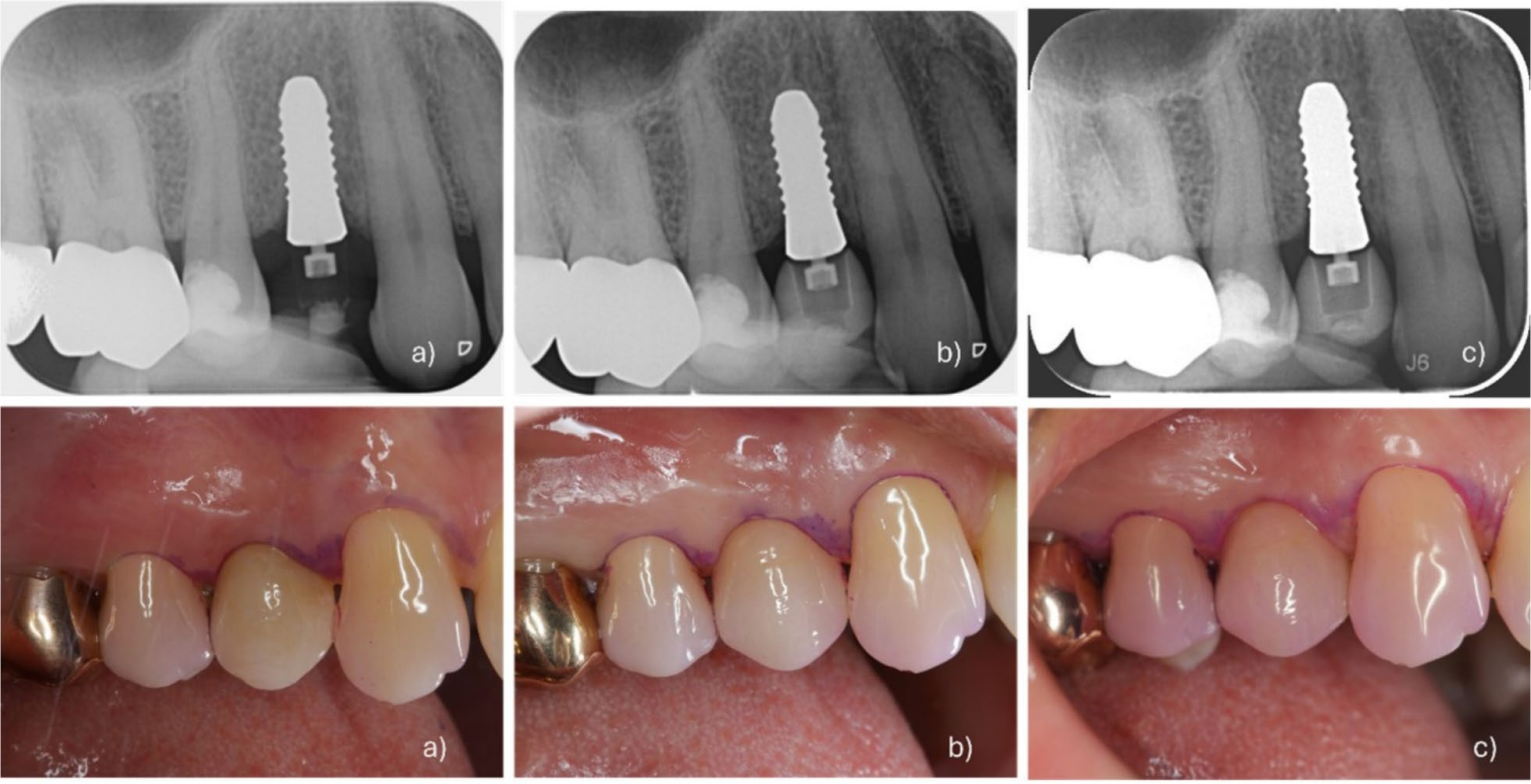
Case of successful implantation and prosthesis placement. X-ray images showing a translucent area around implant abutment is due to the PEKK (Polyetherketoneketone) abutment material, which is radiolucent and thus not visible on x-rays. **a** X-Ray and intraoral photograph at time of provisional prosthesis placement. **b** X-ray and intraoral photograph at time of definitive prosthesis placement. **c** X-Ray and intraoral photograph at 12-month follow-up with stable soft tissue clinical parameters

Twelve months after definitive crown placement the first follow-up visit was scheduled. One case of successful implant placement and prosthetic restoration can be seen in Fig. 3. Three patients were additionally lost for the 12-month follow-up visit. One patient (#2) due to illness and two patients (#5, #7) due to moving to a new location. However, these 3 patients were only lost to the 12-month follow-up visit without implant failure. Telephone interviews confirmed that all implants remained in situ without reported complications. All three patients subsequently attended the 24-month follow-up examination, at which time all implants remained functional without complications. Therefore, these three implants were included in the calculation of the survival rate at the 12-month follow-up (n=14). The oral hygiene level of examined patients can be seen in Table 7.

**Table 7:** Oral hygiene index of examined patients at different visits

Oral Hygiene Index	1	2	3	4	Total number n
At Screening	15	9	0	0	24
At Definitive Loading	7	9	1	0	17
Number of Patients at 12-month follow-up	4	5	2	0	11

There were no technical complications reported. The clinical assessment of PPD and BOP of surviving implants at six sites is summarized in Table 5. At the 12-month follow-up, the 11 examined implants showed a mean PPD of 2.7 ± 0.7 mm. Four sites (6.1%) exhibited PPD > 4 mm and six sites (9.1%) showed PPD > 5 mm. The mean PPD of survived implants increased by 0.3 mm (from 2.4 ± 0.3 mm to 2.7 ± 0.7mm) from definitive loading to the 12-month follow-up. There were no occurrences of suppuration. The survived implants showed an increase of BOP from 17.7% at definitive loading to 26% at 12-month follow-up at the implant sites.

The survival rate for the implants after 12 months was 60.9% (Table 6). Of the 24 initially placed implants, one patient dropped out because he did not attend any follow-up visits. Among the remaining 23 implants, six were lost before definitive loading (two at re-entry and four after provisional loading). An additional three implants were lost by the 12-month follow-up, resulting in a total of 14 surviving implants at that time point. All survived implants after 12 months fulfilled the predefined success criteria leading to a success rate of 60.9% after 12 months. The radiographic evaluation after 12 months showed mean DIB values of 1.4 ± 0.6 mm. The mean DIB of the surviving implants decreased by 0.5 mm from definitive loading to the 12-month follow-up (from 1.9 ± 0.6 mm to 1.4 ± 0.6 mm), indicating an increase of the marginal bone level.

Questionnaire of PROMs for patients with survived implants after 12 months (n = 11) demonstrated the following scores on a 5-point Likert scale (1 = very dissatisfied, 2 = dissatisfied, 3 = neutral, 4 = satisfied, 5 = very satisfied): for comfort, chewing function, taste sensation, fit, and overall satisfaction, the mean score was 4.8 ± 0.4; for aesthetic appearance, the mean score was 4.9 ± 0.3.

## Discussion

This prospective clinical study evaluated the survival rate of two-piece zirconia implants restored with screw-retained ceramic crowns on PEKK abutments over a 12-month period following definitive loading. Our findings revealed an unsatisfactory survival rate, of 60.9% at the 12-month follow-up. The major reasons for implant loss were the lack or loss of osseointegration and implant fracture. Specifically, 2 implants showed mobility at the time of re-entry, 4 implants failed after provisional or definitive loading due to mobility, and 3 implants fractured at the coronal third after loading. These outcomes raise important questions about the factors influencing implant survival in our cohort and underline the need for a critical examination of the potential challenges associated with two-piece zirconia implants in combination with screw-retained restorations.

Comparing the results of this study with existing literature reveals a wide range of survival rates for two-piece zirconia implants, however, all studies reported higher survival rates than 60.9% at 12 months. Cionca et al. [14, 15] reported on 49 initially placed Zeramex T implants, showing survival rates of 87% after one year and 83% after six years. While they did not mention the insertion torque or perioperative antibiotic use, their prosthetic approach differed significantly from ours. They used abutments cemented into the implant with an adhesive resin cement and restored with lithium disilicate glass ceramic crowns cemented in the same way, in contrast to our screw-retained approach. Brull et al. [13] demonstrated a higher survival rate of 96.5% after 3 years with a number of 66 custom-made two-piece zirconia implants. These were inserted with a torque not exceeding 35 N/cm, though perioperative antibiotic use was not specified. Importantly, this study also used cemented abutments, differing from our screw-retained approach. Payer et al. [16], despite a smaller sample size of 16 Ziterion vario T implants, achieved a 93.3% survival rate after 2 years. They used an insertion torque of < 35 N/cm and administered perioperative antibiotics. They bonded ceramic abutments adhesively to the zirconia implants, and placed definitive lithium disilicate restorations. Becker et al. [12] reported on 60 initially placed ZV3 implants, with a 95.8% survival rate after 2 years. While insertion torque was not mentioned, they did use perioperative antibiotics. Notably, 8 of the initially placed 60 implants did not reach primary stability, indicating that lack of osseointegration was also a main problem in their study. Their restoration technique involved cementable glass fibre abutments for cementable crowns, again differing from our screw-retained approach.

Several factors may contribute to the discrepancies between these studies and our findings. Of particular importance are the variations in prosthetic restoration techniques and implant dimensions. Our study utilized screw-retained crowns directly on the implant, which differs significantly from the approaches in other studies. These differences in prosthetic design could substantially influence stress distribution and overall implant performance. For the used implant system, clinicians have the option to choose between titanium and gold screws for abutment fixation. The only study [23] examining one- and two-piece ceramic implants from the same manufacturer with a similar prosthetic workflow found no significant difference in survival rates between one- and two-piece zirconia implants at the implant level. All two-piece zirconia implants were restored with screw-retained crowns on PEKK abutments and gold screws, which is comparable to our prosthetic workflow, differing in the material of the abutment screw (gold screw) and the material of the final crown (resin and porcelain zirconia). The study reported an overall 5-year survival rate for two-piece zirconia implants of 73% at the implant level. Of the four implant failures observed, there was an equal distribution between one-piece and two-piece implants. Notably, all implant losses occurred during the osseointegration phase, prior to the placement of definitive prosthetic restorations. After the final restorations were in place, both implant types demonstrated a 100% survival rate. Although its relatively small sample size (29 implants in 18 patients, with only 9 two-piece implants) limits these findings and highlights the need of further studies, the results may suggest, that the choice of screw material could have had an impact on the stability and longevity of the implant-abutment connections. An important factor influencing the implant-abutment stability is the preload of the abutment screws used, which is also influenced by the screw material. In this context Martin et al. [24] demonstrated, that gold screws achieved higher preload values compared to titanium screws in external hex implants under the same torque conditions. Inadequate preload can lead to screw loosening, which is associated with increased micro-movements at the implant-abutment interface. This stress mismanagement can contribute to unfavorable stress distribution in the implant and surrounding bone, leading to implant fractures or loss of osseointegration [25, 26]. It is important to note that these studies were primarily conducted on titanium implants, and the preload dynamics in ceramic implants, such as the used system, have not been extensively investigated. Further research is needed to fully understand the implications of screw material choice in ceramic implant systems and how it may influence long-term stability and the risk of complications such as screw loosening, implant fracture, or loss of osseointegration.

The lower survival rate observed in our study could potentially be attributed to the unique combination of ceramic implants with screw-retained prosthetic restorations. Unlike previous studies on two-piece ceramic implants that predominantly used cemented abutments, our research employed a screw-retained approach. This methodological difference may have significant implications for stress distribution and overall implant stability. The rigidity of ceramic materials, coupled with the dynamics of a screw-retained system, might lead to stress concentrations that differ from those in cemented systems or titanium implants. Furthermore, the preload achieved with titanium screws in ceramic implants may not provide the same stability as that achieved with gold screws or with titanium screws in titanium implant systems. Other factors, such as differences in implant dimensions, surgical protocols, including insertion torque and use of perioperative antibiotics, may also impact outcomes. The number of implants vary considerably across studies, with our study and some others having small patient numbers, which can affect statistical reliability. These variations in study design and implant characteristics make direct comparisons challenging and highlight the need for more standardized approaches in future research.

Several limitations should be considered when interpreting the results of this study. The relatively small sample size of 24 patients limits the statistical power of the results. Furthermore, this study investigated a specific two-piece zirconia implant system with screw-retained prosthetic restorations on PEKK abutments, which may restrict the generalizability of our results to other zirconia implant systems or prosthetic concepts. Additionally, the absence of a control group with titanium implants makes direct comparison with the current gold standard difficult. As a pilot study, the results should be confirmed by larger multicenter studies to validate the findings and expand the available clinical data.

## Data Availability

The datasets used and/or analysed during the current study are available from the corresponding author on reasonable request.

## Abbreviations

BOP: Bleeding on probing
CBCT: Cone-beam computer tomography
DICOM: Digital imaging and communications in medicine
DIB: Distance implant shoulder to first bone contact at implant
DKRS: Deutsches Register klinischer Studien
IC: Informed consent
MGI: Modified gingival index
MIC: Munich implant concept
PEKK: Polyetherketoneketone
PPI: Plaque assessment
PPD: Probing pocket depths
PROMs: Patient-reported outcome measures
STL: Standard tessellation language
Y-TZP: Yttrium-stabilized tetragonal polycrystalline zirconia
